# Biomechanical response of lumbar facet joints under follower preload: a finite element study

**DOI:** 10.1186/s12891-016-0980-4

**Published:** 2016-03-15

**Authors:** Cheng-Fei Du, Nan Yang, Jun-Chao Guo, Yun-Peng Huang, Chunqiu Zhang

**Affiliations:** Tianjin Key Laboratory for Advanced Mechatronic System Design and Intelligent Control, Tianjin University of Technology, No. 391, Binshui West Road, Xiqing District, 300384 Tianjin, China; School of Biological Science and Medical Engineering, Key Laboratory for Biomechanics and Mechanobiology of Ministry of Education, Beihang University, 100191 Beijing, China; Department of Orthopaedics, First Affiliated Hospital of Fujian Medical University, 350005 Fuzhou, China

**Keywords:** Facet joint, Follower load, Biomechanics, Lumbar spine, Finite element

## Abstract

**Background:**

Facet joints play a significant role in providing stability to the spine and they have been associated with low back pain symptoms and other spinal disorders. The influence of a follower load on biomechanics of facet joints is unknown. A comprehensive research on the biomechanical role of facets may provide insight into facet joint instability and degeneration.

**Method:**

A nonlinear finite element (FE) model of lumbar spine (L1-S1) was developed and validated to study the biomechanical response of facets, with different values of follower preload (0 N,500 N,800 N,1200 N), under loadings in the three anatomic planes. In this model, special attention was paid to the modeling of facet joints, including cartilage layer. The asymmetry in the biomechanical response of facets was also discussed. A rate of change (ROC) and an average asymmetry factor (AAF) were introduced to explore and evaluate the preload effect on these facet contact parameters and on the asymmetry under different loading conditions.

**Results:**

The biomechanical response of facets changed according to the loading condition. The preload amplified the facet force, contact area and contact pressure in flexion-extension; the same effect was observed on the ipsilateral facet while an opposite effect could be seen on the contralateral facet during lateral bending. For torsion loading, the preload increased contact area, decreased the mean contact pressure, but had almost no effect on facet force. However, all the effects of follower load on facet response became weaker with the increase of preload. The greatest asymmetry of facet response could be found on the ipsilateral side during lateral bending, followed by flexion, bending (contralateral side), extension and torsion. This asymmetry could be amplified by preload in the bending (ipsilateral), torsion loading group, while being reduced in the flexion group.

**Conclusions:**

An analysis combining patterns of contact pressure distribution, facet load, contact area and contact pressure can provide more insight into the biomechanical role of facets under various moment loadings and follower loads. The effect of asymmetry on facet joint response should be fully considered in biomechanical studies of lumbar spine, especially in post structures subjected to physiological loadings.

**Electronic supplementary material:**

The online version of this article (doi:10.1186/s12891-016-0980-4) contains supplementary material, which is available to authorized users.

## Background

As a part of the three-column structure of vertebrae, facet joints play a significant role in maintaining the stability of spinal motion. Facets transfer load through spinal column and restrict the motion of vertebrae, especially in the direction of extension and rotation [1–3]. A partial or full facetectomy may clearly decrease the mechanical stiffness of the motion segment [4]. Also, changes in the mechanical environment of facet joints have been associated with osteoarthritis or degeneration, which could eventually lead to low back pain [5–7]. Thus, a better understanding of the role of facets in spine biomechanics may provide insight into facet joint instability and degeneration.

A number of experimental studies have been conducted to investigate the facet loading parameters under external loads [2, 8, 9]. The magnitude of load transmission through the facet joints has been reported to range between 9 and 57 % for L2-L3 level and 8–28 % for L4-L5 level under axial compression [9]. The moment in both extension and axial rotation produce large facet loads but in flexion, facet load has been shown to be minimal [2, 10]. Goel et al. [11] found that the load was distributed among the right facet, the disk, the left *ligamentum flavum* and the left capsular ligament during right lateral bending. They also showed that the addition of a preload reduced the load in these spinal elements, except for the disk. For surgically altered spinal segments, the facetectomy causes a significant decrease of facet force and contact area in the remaining facet [8]; and the dynamic posterior stabilization does increase peak facet contact forces during flexion and lateral bending, while it does not affect these loads during extension or axial rotation [12]. The effect of interspinous implant on the facet loading parameters such as force, contact area and pressure at the implanted and adjacent levels has also been studied [13].

On the other hand, analytical studies, such as finite element (FE) method, have been widely conducted to quantify the biomechanical characteristic of facet joint. For example, Sharma [14] investigated the role of facets in load transmission and its sensitivity to facet geometric parameters. In another FE study, Wang et al. [15] found that posture affected facet forces compared with the loading rate. In a study by Teo et al. [16], an anatomically accurate and validated FE model of the human lumbar L2–L3 motion segment was developed and tested under axial compressive loading, to investigate the role of normal and degenerated facet joints in load-bearing. Schmidt et al. [17] explored the effect of multilevel lumbar disc arthroplasty on spine kinematics and facet joint loads during flexion and extension. Kuo et al. [18] investigated whether the asymmetric response of facet joints was amplified by the loadings in various postures.

Follower load is defined as the compressive load directed approximately along the axis of the spine as the result of the trunk muscle action and its value is often beyond 1000 N [19]. Obviously, the application of large follower load can more realistically reflect the physiological loading. The application of follower load during in vitro experiment and FE study were first presented by Patwardhan et al. [19] and Shirazi-Adl et al. [20] respectively. Then, the effects of follower load on lumbar spine biomechanics have been investigated, such as load-carrying capacity, intradiscal pressure and intersegmental rotation [21, 22]. However, to the authors’ knowledge, no investigator has addressed the influence of follower load on the biomechanical behavior of facet joint.

The limitations noticed in the foregoing literature review can be summarized as follows: most studies that addressed the biomechanics of facet joint were only focused on single motion segment and the loadings were often applied in only one or two directions [2, 3, 8–10, 14–16, 23]. In some studies, the preload was often applied with low magnitude and therefore cannot sufficiently represents the real physiological loading condition [10–12, 17]. For the FE studies, most facet cartilages were assigned with a uniform thickness, or even simplified as gap elements or contact elements which cannot realistically and sufficiently predict the complex behavior of facets during contact [1, 3, 14–18, 20, 22, 23]. Moreover, most articular surfaces of facets were assumed to be planar and parallel with gap [1, 3, 14, 15, 17, 20, 23]. In fact, the cartilage surfaces are curved and the thickness of the facet cartilage varies according to the vertebral level and the locations within a same level [24–26]. A more geometrically realistic model of facet can help us to further confirm the biomechanical response of facet joints under various physiological loadings. Finally, linear material constitutive representation was used in model development in much of these studies [3, 14, 16, 18]. Nonlinear modeling can obtain more realistic mechanical behavior of facet joint since obvious nonlinear responses of lumbar spine have been reported in a great number of experimental studies [10, 27–29].

Accordingly, the aim of this study was to investigate the biomechanical response of facet joints under follower load in the three anatomic planes. To realize it, a three-dimensional nonlinear FE model of the complete lumbar spine (L1-S1) was developed and validated. In this study, a graded follower preload with increasing values of 0 N, 500 N, 800 N, 1200 N was imposed on lumbar spine to explore its effect on the biomechanical behavior of facet joints with regard to contact force, contact area, mean contact pressure and pressure distribution. The difference in facet biomechanics between the left and the right sides was included in this study. We also determined how this asymmetry was affected by various loading conditions.

## Method

### Development of the model

**Fig. 1:**
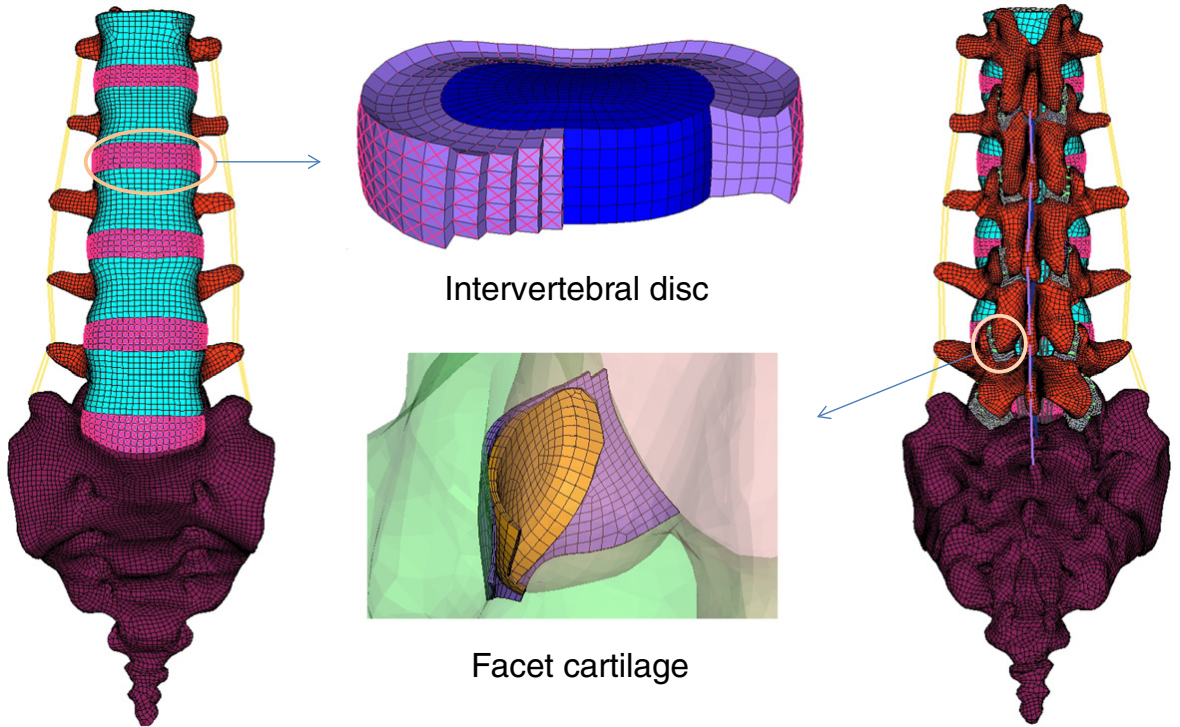
FE model of lumbar spine with the details of the disc and the facet cartilage

The procedures for developing the FE model of lumbar spine and the materials used in this model were introduced in the author’s previous study on the response of spine under dynamic loading [30]. Briefly, a nonlinear FE model of complete lumbar spinal segments (L1-S1) was generated based on geometrical reconstruction from computer tomography (CT) scan. The image segmentation and reconstruction of geometrical model were finished in a medical image processing software (Mimics 10.0; Materialise Technologies, Leuven, Belgium) and a reverse engineering and scanning software (Geomagic studio 10.0; Geomagic Inc., North Carolina, USA), respectively. The mesh was generated in a CAE pre-processoring software (Hypermesh 11.0; Altair Engineering Corp, Michigan, USA). A vertebra consists of a cortical wall, a cancellous bone, endplates and post elements. The intervertebral disc was made up of the nucleus and annulus, and the annulus was assumed to be composed of a ground substance reinforced by a collagen fiber network. The fibers embedded in the ground substance were oriented at an average angle of ±30° to the endplates. The seven ligaments: the anterior (ALL) and posterior (PLL) longitudinal ligaments, the intertransverse (ITL), flavum (FL), supraspinous (SSL), interspinous (ISL) and capsular (CL) ligaments were included. Facet cartilage was represented by three layers of hex elements with inhomogeneous thickness and the gap between the facet articular surfaces was about 0.1 mm. The distribution of cartilage thickness in each facet was in accordance with the findings of Woldtvedt et al. [24, 26]. The complete FE model of lumbar L1-S1 segments is shown in Fig. 1.

All the components of vertebrae were considered as isotropic homogeneous elastic materials. The annulus ground substance, nucleus pulposus and facet cartilage were simulated to be nearly incompressible and hyper-elastic [28, 30]. Ligaments and fibers were simulated as tension-only spring with nonlinear properties according to the research of Shirazi-Adl [31]. All the material properties and elements information (types and number) for each parts of this lumbar model were listed in Table 1.

**Table 1:** Material properties and elements information of the model used in this study

Components name	Young’s modulus (MPa)	Poisson’s ratio	Element type	Element no.
Cortical bone	14000	0.30	Hex	2585
Cancellous bone	100	0.2	Tetra	129931
Posterior elements	3500	0.25	Tetra	250978
Endplate	10000	0.25	Hex	4921
Sacrum	5000	0.2	Tetra	200295
Facet cartilage	Neo-Hookean, C10 = 2		Hex	7293
Annulus	Mooney–Rivlin, C1 = 0.18, C2 = 0.045		Hex	6000
Nucleus pulpous	Mooney–Rivlin, C1 = 0.12, C2 = 0.03		Hex	7200
Fiber	Calibrated stress–strain curves		Spring	14400
Ligament	Calibrated deflection–force curves		Spring	234

### Calibration and validation

Before using this model to study the biomechanical response of facets, its calibration and validation had to be operated. The calibration procedure was conducted according to the method presented by Schmidt et al. [32, 33]. The calibration factors of collagen fibers and ligaments were varied in order to obtain the optimal values (i.e., for which the range of motion (ROM) predicted by the model well matched the in vitro experimental results).

Validation was then undertaken by comparing the predicted data obtained by the current model with the results from the literature. The range of motion of each segment under moment loading in the three anatomic planes and the disc compression under a follower load of 1200 N were calculated and compared with the experimental and simulated data presented by Renner et al. [22]. The boundary conditions and loading were set to replicate the in vitro experiment. The surface-to-surface contact between facet joints was defined as frictionless during the entire validation simulation, as well as for the following biomechanical study of facet. All the simulation works were conducted in a commercial finite element package (Abaqus 6.11; Dassault Systèmes Simulia Corporation, Pennsylvania, USA).

### Biomechanical study of facet

The study was designed in two steps. In step 1, a pure moment of 7.5 N · m in different successive postures (flexion, extension, right bending, left bending, right torsion, left torsion) was imposed on a node which was coupled with the upper endplate of L1. In step 2, an additional follower preload of 500 N, 800 N and 1200 N was applied respectively on each segment and for each loading case to determine the effect of the preload on the facet joint. The bilateral sacro-iliac joint surfaces were constrained in all directions during loading. The contact variables including the resultant contact force, the contact area, the mean contact pressure (i.e., the contact force divided by the contact area) and the distribution of contact pressure of each facet in each loading case were recorded and compared.

To further investigate the effect of an increased preload on the facet response, a rate of change (ROC) was introduced. This ROC was defined as the percent change in facet response parameters such as the contact force, the contact area and the mean contact pressure, relative to their baseline values in a pure moment condition, per increment of 100 N preload. An average asymmetry factor (AAF) was also introduced to quantify the asymmetry existing in facet response under various loading conditions. AAF was calculated as the difference in facet response between the two sides, divided by the lower value of this response parameter. For lateral bending and torsion postures, the difference between ipsilateral or contralateral facet was used. For example, when considering the study of the contact force during bending, the AAF for contralateral facet equaled to the facet force values on the left side minus the values on the right side, and then divided by the right side values, considering that the right facet carried smaller contact force than the left facet. When the value of contact parameter was equal to 0, an AAF of 1000 was assumed.

The method consisting in applying the follower load can be found elsewhere [34]. Connector elements between each pair of endplates were created and connected each other one by one. The endpoints of these connectors, coupled with one surface of endplate, and their location were close to the center of each endplate (Fig. 1). To minimize the additional impact of connectors on the lumbar model, a small stiffness of 1 N/mm was assumed for the connector. The connector load was exerted to apply the follower load. Thus, the force line of the follower load could pass through the center of rotation of each segment and then almost follows the curvature of the lumbar spine.

## Results

### Calibration and validation

**Fig. 2:**
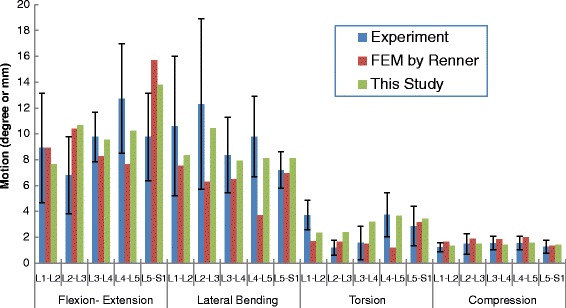
Comparison of predicted results by the current FE model against investigation by Renner [22]

The optimized calibration factors of fibers and ligaments were as follows: fibers, 0.49; ALL, 1.0; PLL, 0.3; ITL, 1.0; FL, 5.0; SSL, 0.07; ISL, 0.08 and CL, 5.0. With these calibrated material properties, the biomechanical response of the lumbar model was expected to be closer from the in vitro results. The validation results are shown in Fig. 2 and Additional file 1. It was found that the motion predicted by the present model compared well with the results of in vitro and other FE studies, except for the L2-L3 level, where a slightly higher ROM was seen. Accordingly, this model could be further used for the study of the biomechanical response of lumbar spine facet joints under various follower loads and moments.

### Facet force

**Fig. 3:**
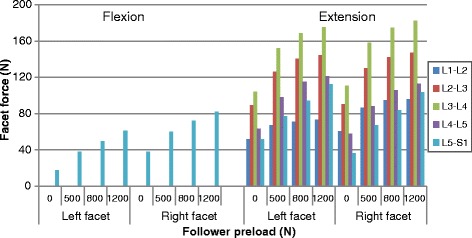
Predicted facet forces at different levels under various preload in flexion and extension postures

**Fig. 4:**
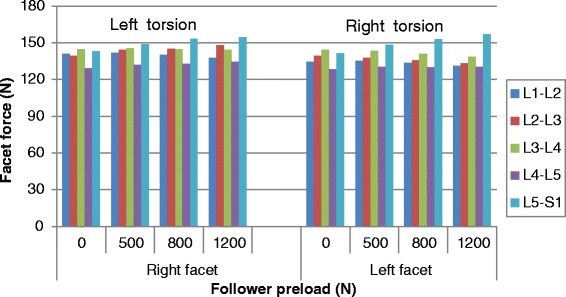
Predicted facet forces at different levels under various preloads in torsion posture

**Fig. 5:**
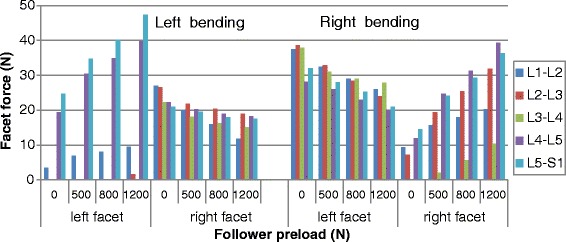
Predicted facet forces at different levels under various preloads in lateral bending posture

The resultant facet contact force at various lumbar levels and under various follower preload from 0 to 1200 N and various moments are shown in Figs. 3, 4 and 5 (Raw data can be found in Additional file 2). On the left side, the facet joints carried 18.1 N at the L5-S1 level and no contact force at other levels under a pure flexion moment of 7.5 N m, whereas they carried 51.6 N, 89.5 N, 115.9 N, 63.5 N and 51.86 N respectively from L1-L2 to L5-S1 levels, during extension. The loads transmitted through facet at middle levels (L2-L3 and L3-L4) were higher than those at other levels. The addition of follower load increased facet force at all levels, and more preload caused more increment in facet force. For example, the contact force in the left facet at the L4-L5 level increased by 54.3 %, 81.3 %, 90.9 % respectively when preload was 500 N, 800 N and 1200 N. A similar tendency was also found in the right facet. It can also be noticed that the facet forces on the right side were slightly different from those of the left side, this asymmetry being affected by the preload (Fig. 3). For example, at the L4-L5 level, the difference in contact force between the two sides was 5.5 N without preload, this difference changing to 8.4 N with a preload of 1200 N.

The pure torsion moment produced facet force only in the ipsilateral facet joint and the force values were obviously greater than those observed in flexion-extension moment, as shown in Fig. 4. The facet forces at the different levels ranged from 137.7 N to 141 N for the left facet and from 131.0 N to 134.5 N for the right facet. The application of preload almost had no effect on the facet force for the two sides and at all levels. A very little asymmetry between the two sides could be found in torsion.

However, a large asymmetry existed under a bending posture loading (Fig. 5). At first, in the ipsilateral facet joint, the number of levels carrying load under left bending loading was less than it under right bending loading. For example, under pure moment, this concerned three levels during left bending while it concerned four levels during right bending. Secondly, in the contralateral facet joints, the facet loads in the left bending were obviously higher than those in right bending for all the preload conditions, while in the ipsilateral facet joints, the contrary was observed. Application of a follower preload increased the ipsilateral facet force, but decreased the contralateral facet force at all levels. For example, when considering the L4-L5 facets, the preload increased the facet contact force by 57.2, 79.8 and 105.1 % on the left side and decreased this value by 9.0, 14.9 and 18.0 % on the right side when the follower load was increased up to 1200 N.

### Contact area

**Fig. 6:**
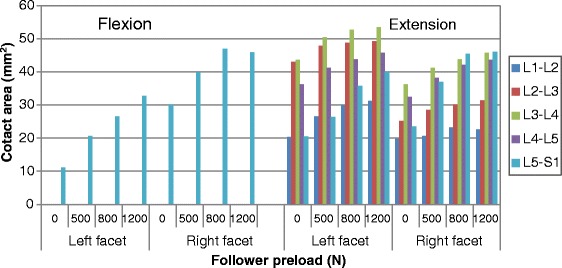
Predicted facet contact areas at different levels in flexion and extension postures under various preloads

**Fig. 7:**
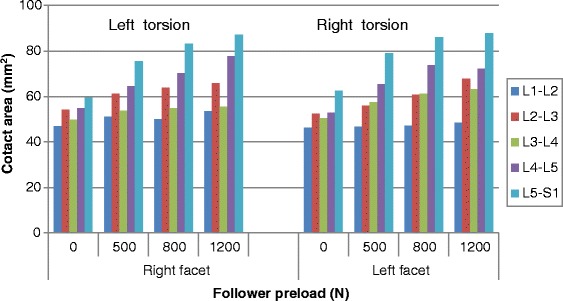
Predicted facet contact areas at different levels in torsion postures under various preloads

**Fig. 8:**
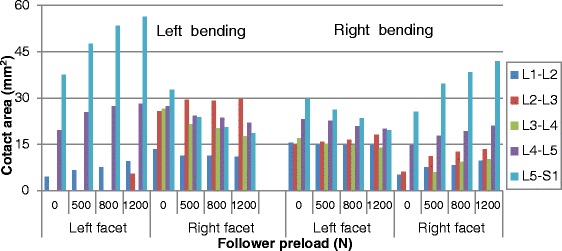
Predicted facet contact areas at different levels in lateral bending postures under various preloads

The facet contact area at all levels under various moments and preloads are shown in Figs. 6, 7 and 8 (Raw data can be found in Additional file 2) . Similarly to the facet force results, the preload had an amplified effect on force contact area at all levels (i.e., from L1-L2 to L5-S1) during flexion-extension (Fig. 6). During bending, the preload had the same influence on facet contact area for the two sides as it had on facet force (Fig. 8). However, the facet of the lower levels produced more contact area in contralateral facet joints, which was different from the facet force results. In torsion posture, the increased effect of preload on contact area could be clearly observed, which was also different from its effect on contact force. In addition, more asymmetry existed in the analysis of contact area under various loadings. For example, during extension, facet contact area at the L2-L3 level on the right side was far less than on the left side, while this was not the case when considering facet force.

### Mean contact pressure

**Fig. 9:**
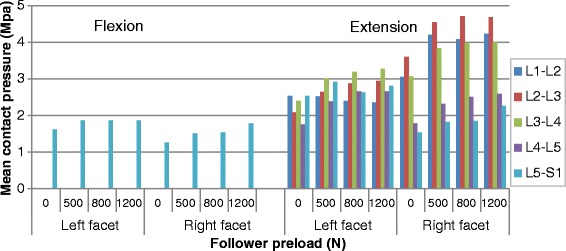
Predicted mean contact pressures at different levels in flexion and extension postures under various preloads

**Fig. 10:**
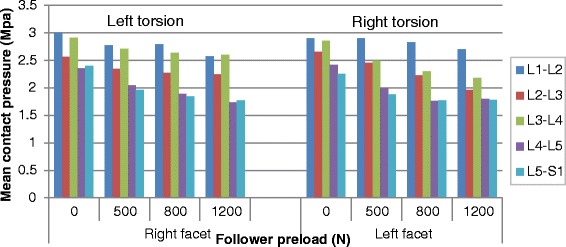
Predicted mean contact pressures at different levels in torsion posture under various preloads

**Fig. 11:**
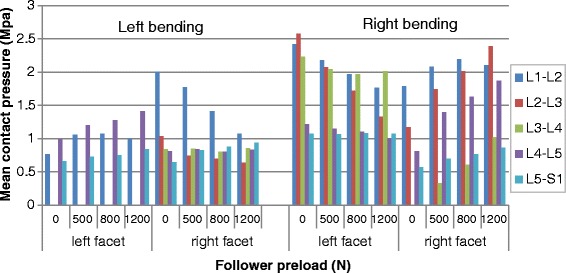
Predicted mean contact pressures at different levels in lateral bending postures under various preloads

The calculated mean facet contact pressures in different loading conditions are shown in Figs. 9, 10 and 11 (Raw data can be found in Additional file 3). It could be noticed that the variation in mean contact pressure with the preload showed a trend similar to the variation in contact force and contact area on the sagittal or coronal plane. However, the preload reduced the mean contact pressure in torsion (Fig. 10), compared to none effect of preload on contact force and an increase effect of preload on contact area, in the same posture.

**Fig. 12:**
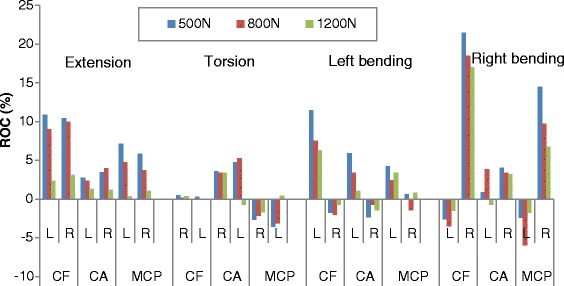
ROC (rate of change) of facet contact force, contact area, mean contact pressure at the L4-L5 level in different loading conditions. L: left facet; R: right facet; CF: contact force; CA: contact area; MCP: mean contact pressure

In the following study on the effect of preload on facet, the rate of change (ROC) was introduced. This variable was defined as the percent change in facet response parameters, relative to the baseline values in a pure moment condition, per increment of 100 N preload. The ROC results indicated that the absolute value of ROC (considering that the decrement of response parameters would produce a negative value) showed an overall decrease when the preload increased. For example, when considering the L4-L5 level, the ROC of contact force during extension was 10.9 and 10.4 % respectively for the left and right sides with a preload of 500 N, while these two values changed to 9.0, 9.9 and 2.4 %, 3.1 % with a preload of 800 N and 1200 N respectively (Fig. 12). This means that the effect of preload on facet contact force became smaller with the increment of preload. A similar trend was also found in contact area and contact pressure in other postures.

Table 2 shows the changes in AAF, with or without preload, under various moment loadings. Clearly, an asymmetry existed for different postures, ranking as follows, from the largest to the smallest asymmetry: bending (ipsilateral), flexion, bending (contralateral), extension and torsion (contralateral). The preload had an obvious amplification effect on facet responses in bending (ipsilateral), torsion (contralateral), while a reduction effect was found on facet responses in flexion. The increment of preload had a slight influence (increase or decrease) on the facet contact parameters during extension and bending (contralateral).

**Table 2:** Average asymmetry factor (AAF) under various moments and preloads

	Flexion			Extension			Bending(ipsilateral)			Bending(contralateral)			Torsion(contralateral)		
	CF	CA	MCP	CF	CA	MCP	CF	CA	MCP	CF	CA	MCP	CF	CA	MCP
0	110.0	169.9	28.5	15.5	24.2	37.7	259.9	223.4	577.5	46.6	43.1	84.1	1.4	3.1	3.6
500	56.7	93.7	23.6	12.4	33.3	45.9	438.4	423.6	799.2	51.4	53.6	83.4	2.5	6.3	5.0
800	45.2	76.7	21.7	12.3	28.1	41.8	433.9	422.4	831.8	52.7	50.9	74.9	3.4	6.2	5.8
1200	34.2	39.9	4.2	10.3	26.1	37.4	385.8	313.4	810.7	52.1	32.5	56.1	5.0	7.2	8.6

(*CF:* contact force; *CA:* contact area; *MCP*: mean contact pressure)

### Distribution of contact pressure

**Fig. 13:**
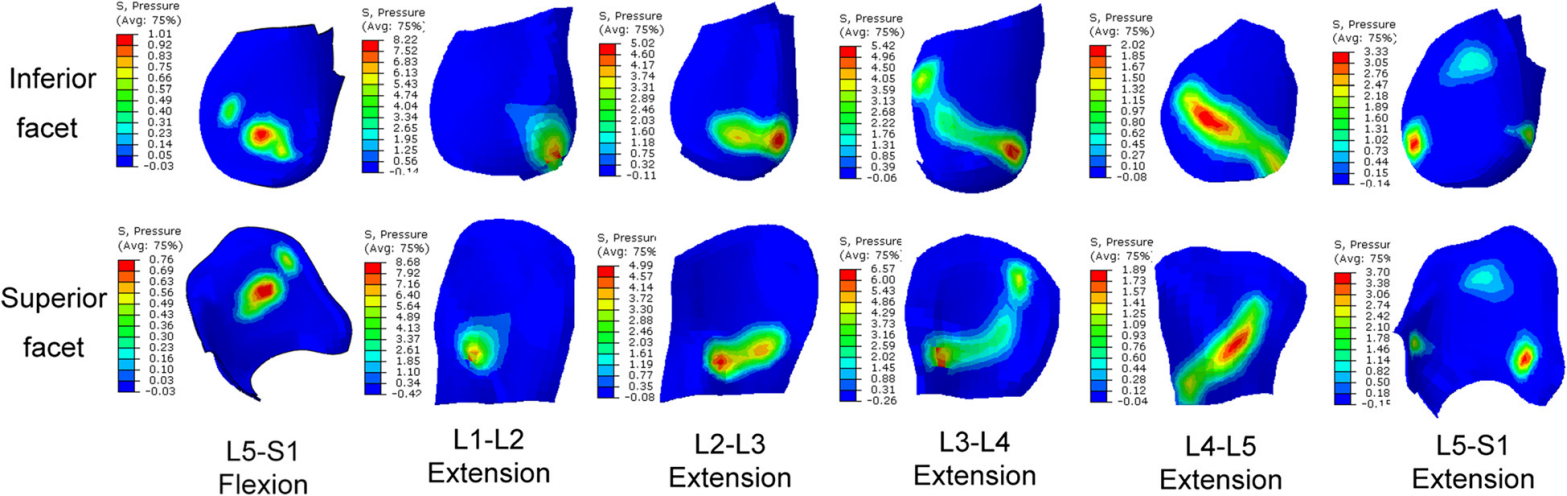
Distribution of facet contact pressure at different levels under flexion and extension loadings

**Fig. 14:**
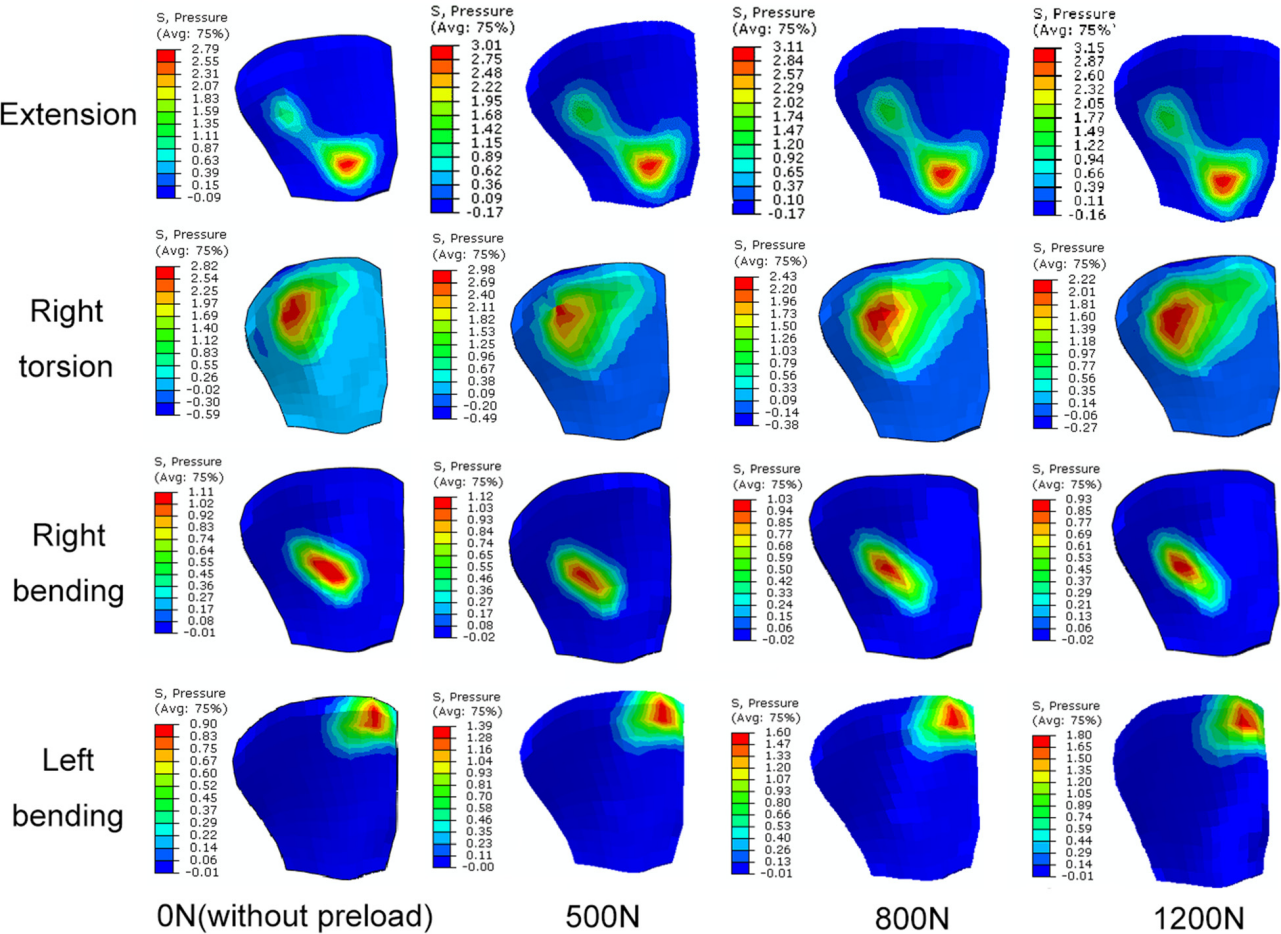
Distribution of contact pressure on the superior left L5 facet cartilage under various follower preloads

Figure 13 shows the distribution of facet contact pressures at various levels under flexion-extension moment. Analysis of the results revealed that the contact occurred between the upper edge of the inferior facet and the middle regions of the adjacent superior articular surface in flexion, while the contact area was mainly located in the lower region of the inferior facet surface and the lower margin of the superior facet. Moreover, the contact area gradually expanded to the lateral region in the lower levels. In the bending and torsion loading conditions, the articular surface bore fully different pattern of pressure distribution from the one observed in flexion-extension loading. For example, when considering the superior left facet of L5, the contact pressures were concentrated in the mid area for the contralateral joint and in the upper edge for the ipsilateral joint under bending loading and mid and upper region (Fig. 14). When applying a preload of 1200 N, the peak contact pressure in superior left facet of L5 changed from 2.79Mpa to 3.15Mpa in extension and from 0.9Mpa to 1.8Mpa in left bending loading, while it was reduced from 2.82Mpa to 2.22Mpa during right torsion and from 1.11Mpa to 0.93Mpa during right bending. This variation tendency was the same as for the mean contact pressure, as shown in Figs. 9, 10 and 11.

## Discussion

A three-dimensional non-linear FE model of lumbar spine has been developed and validated to evaluate the full biomechanical behavior of the facet joints, with or without follower preload, under various moment loadings. The complex geometry of each vertebra especially the facet joints and the facet cartilage with inhomogeneous thickness and curved surface (implemented according to in vitro measurements) all made this model more realistic and the results more accurate. The motion of each segment predicted by the present model compared well with the results of in vitro and other FE studies. The method used in this study and consisting in applying a follower load has been proved to successfully produce a large follower preload with minimal rotation at each level of the lumbar spine. Therefore, the present model could be used to study the effect of preload on the biomechanical properties of facet such as facet force, facet contact area and contact pressure distribution in different postures.

The influence of preload on facet joint has been studied by few researchers [11, 12, 18], who only focused on one motion of segment, with a low preload (less than 600 N). The in vivo follower load often exceeds 1000 N and the way this follower load affects load-carrying capacity, intradiscal pressure and intersegmental rotation of lumbar has been investigated [19, 35]. The study on facet joint under large follower preload can provide more insight into the biomechanical role of facet under physiological loadings and could be a supplement to research on follower load.

Because the biomechanical responses of facet joint are sensitive to loading and boundary conditions, measuring method and geometric parameters (orientation, area and initial joint gap) and so on, considerable divergence was exhibited among experimental and FE studies on facet of lumbar spine. For example, when considering the loading during extension, Buttermann et al. [36] reported a facet load of 74 N at the L2-L3 level under a moment of 2 Nm. However, Goel et al. [11] noticed a value of 52 N at the same level under a moment of 6.9 N m and a value of 27 N at the L3-L4 level under a moment of 7.5 N m was reported by Zhu et al. [37]. The predicted facet force under extension and torsion loadings appears to agree with the results of Kuo et al. [18], Shealy et al. [6] and Woldtvedt et al.[26]. But our predicted facet force seems to be larger than the values obtained by Zhu et al. [37], Niosi et al. [12] and Schmidt et al. [17]. A possible reason that could explain this discrepancy is that, in experimental studies, a part of the facet forces was not identified due to impingement, and the thickness, orientation and material of facet cartilage assumed in other FE studies were different from those used in this model. The findings that torsion and extension moments generate large contact forces while lateral bending and flexion generate small contact force (Figs. 3, 4 and 5) in this study are consistent with the results from other researches [3, 14, 17, 25, 37]. It should be noted that flexion loading generated contact force only at the L5-S1 level and its magnitude was 18 N and 38.6 N respectively for the left and right sides. The difference existing in the curvature of each motion segment, the volume of the vertebral disc and the geometry of facet joint all contribute to the variability of facet force at different levels. For example, the L2-L3 and L3-L4 levels carried larger facet loads than other levels in this study and similar results have been reported in other studies [1, 18].

An obvious increase in facet force due to a compressive preload in extension posture is observed in Fig. 3. This result can be explained as follows: the compressive follower load makes the upper part of superior facet move away from the inferior facet surface but the lower part of superior facet move towards the inferior facet surface due to the complex geometry of articular surface in this model. However, the contact area on the superior facet surface is mainly located in the lower margin (Fig. 13). The nonlinear material properties of facet cartilage and disc make the compressive displacement of superior facet become smaller with the same increment of preload. Furthermore, the deformation of disc may induce a change of direction of the follower load, which may in turn change the relative motion of facet articular surfaces and the contact status. Therefore, the preload amplified both facet force and contact area during extension, but this amplification effect became smaller as the preload increased (Fig. 12). The smaller increase in contact area than in facet force causes both mean and peak contact pressure to become higher with the increase of preload (Figs. 12 and 14). The variation of facet force, contact area and contact pressure during lateral bending and torsion can also be explained by the patterns of contact and the motion of superior facet relative to inferior facet under moment and preload. Therefore, the overall evaluation of biomechanical behaviors of facet relating to contact pressure distribution, contact load, contact area and contact pressure can provide more insights into the effect of follower preload on facet and load transmission through facet joint.

The asymmetry between the left and right facet joints was also investigated in this study. Although this asymmetry phenomenon has been considered in previous studies investigating the biomechanics of spine [1, 9, 12, 18, 38], none of them has addressed the effect of loading conditions on asymmetry. The asymmetry existing in the structure of post elements may cause the asymmetry of facet response. As found in this paper, the prediction of facet force, contact area and contact pressure demonstrated a great asymmetry in ipsilateral facet under lateral bending moment, followed by flexion, bending (contralateral), extension and torsion (Table 1). The addition of a preload provided very different effects of asymmetry for different contact parameters under different loading conditions. For example, all the facet contact parameters increased in bending (ipsilateral) and torsion loading group, while them decreased in flexion group with the increment of preload. However, for the same loading group such as extension, the contact force decreased while a slight increase of contact area and mean contact pressure was found with the preload increase. These findings revealed the importance of considering asymmetry in biomechanical studies of lumbar spine, especially in post structures. The tendency described in the present study and related to the effect of preload on facet force during torsion is slightly different from another study [18], in which the asymmetry was obviously affected by preload only in left torsion but not in right torsion. This disparity is partly due to the difference in the modeling of facet joint and the material used between the two models.

There were several inherent limitations in this study. The geometry of facet cartilage layer was extracted from previously published in vitro measurements and there was probably a difference between the cartilage model used in this study and its real anatomy information. Future improvement in the development of FE model should be made by combining the data from MRI and CT scans from a same subject. In addition, the ligaments were modeled as one-dimensional nonlinear springs in this study, which cannot represent the real geometry. A greater accuracy should be considered in future work. This model was developed based on the geometry of individual lumbar spine and it can only be used to reflect the trends in the responses of the lumbar spine under various loading conditions.

## Conclusion

In conclusion, the results indicated that the follower preload made the facet force, contact area and contact pressure increase during flexion-extension, while it made these parameters increase for ipsilateral facet and decrease for contralateral facet during lateral bending. The facet force barely changed with the preload, while the contact area may be extended and the contact pressure became smaller during torsion loading. The effect of follower preload on facet response became weaker with the preload increase. The most obvious asymmetry of the biomechanical responses occured during lateral bending (ipsilateral), followed by flexion, bending (contralateral), extension and torsion. This asymmetry could be amplified by a preload during bending (ipsilateral) and torsion loading, whereas it could be reduced during flexion. Overall, the evaluation of contact pressure distribution, facet load, contact area and contact pressure can provide more insight into the biomechanical role of facet under various moment loadings and follower loads.

### Availability of data and materials

The datasets supporting the conclusions of this article are included within the article and its additional files.

## Additional files

Additional file 1: Raw data of validation for the lumbar spine model. (XLSX 16 kb) Additional file 2: Raw data of prediected facet forces/contact areas in different postures. (XLSX 37 kb) Additional file 3: Raw data of prediected mean contact pressures in different postures. (XLSX 26 kb)

## Abbreviations

AAF: average asymmetry factor
ALL: anterior longitudinal ligament
CA: contact area
CF: contact force
CL: capsular ligament
FE: finite element
FL: flavum ligament
ISL: interspinous ligament
ITL: intertransverse ligament
MCP: mean contact pressure
PLL: posterior longitudinal ligament
ROC: rate of change
SSL: supraspinous ligament
